# CT findings and clinical characteristics in distinguishing renal urothelial carcinoma mimicking renal cell carcinoma from clear cell renal cell carcinoma

**DOI:** 10.1186/s12894-023-01393-5

**Published:** 2024-01-03

**Authors:** Xin Chen, Xiao Feng, Yidi Chen, Fulin Huang, Liling Long

**Affiliations:** 1 Department of Radiology, The First Affiliated Hospital of Guangxi Medical University, No.6 Shuangyong road, Nanning, Guangxi 530021 China; 2 Department of Radiology, Jiangjin Hospital of Chongqing University, No.725, Jiangzhou Avenue, Dingshan Street, Chongqing, 402260 China; 3 Department of Radiology, West China Hospital, Sichuan University, 37 Guoxue alley, Chengdu, Sichuan 610041 China

**Keywords:** Renal urothelial carcinoma, ccRCC, Computed tomography, Collecting system invasion, Differential diagnosis, Predictive model

## Abstract

**Background:**

We aimed to characterize the clinical and multiphase computed tomography (CT) features, which can distinguish renal urothelial carcinoma (RUC) mimicking renal cell carcinoma (RCC) from clear cell renal cell carcinoma (ccRCC) with collecting system invasion (CSI).

**Methods:**

Data from 56 patients with RUC (46 men and 10 women) and 366 patients with ccRCC (262 men and 104 women) were collected and assessed retrospectively. The median age was 65.50 (IQR: 56.25–69.75) and 53.50 (IQR: 42.25–62.5) years, respectively. Univariate and multivariate logistic regression analyses were performed on clinical and CT characteristics to determine independent factors for distinguishing RUC and ccRCC, and an integrated predictive model was constructed. Differential diagnostic performance was assessed using the area under the receiver operating characteristic curve (AUC).

**Results:**

The independent predictors for differentiating RUC from ccRCC were infiltrative growth pattern, hydronephrosis, heterogeneous enhancement, preserving reniform contour, and hematuria. The differential diagnostic performance of the integrated predictive model-1 (AUC: 0.947, sensitivity: 89.07%, specificity: 89.29%) and model-2 (AUC: 0.960, sensitivity: 92.1%, specificity: 89.3%) were both better than that of the infiltrative growth pattern (AUC: 0.830, sensitivity: 71.9%, specificity: 92.9%), heterogeneous enhancement (AUC: 0.771, sensitivity: 86.3%, specificity: 67.9%), preserving reniform contour (AUC = 0.758, sensitivity: 85.5%, specificity: 66.1%), hydronephrosis (AUC: 0.733, sensitivity: 87.7%, specificity: 58.9%), or hematuria (AUC: 0.706, sensitivity: 79.5%, specificity: 51.8%).

**Conclusion:**

The CT and clinical characteristics showed extraordinary discriminative abilities in the differential diagnosis of RUC and ccRCC, which might provide helpful information for clinical decision-making.

**Supplementary Information:**

The online version contains supplementary material available at 10.1186/s12894-023-01393-5.

## Introduction

Clear cell renal cell carcinoma (ccRCC), as the most common renal malignant tumor [1], accounts for 70–80% of renal cell carcinomas (RCC) [2, 3]. Most cases of ccRCC are present with solid masses in the renal parenchyma or perirenal fat and the renal pelvis occasionally, while a few ccRCCs are present with cystic-dominant masses. Upper urinary tract urothelial carcinoma (UUTUC) accounted for 5–10% of urothelial carcinoma (UC) [4]. It was reported that renal urothelial carcinomas (RUC) were more common than ureteral UC [5]. Due to their distinct therapeutic regimens (radical nephrectomy and radical ureteral nephrectomy), the accurate differential diagnosis before the operation was warranted for urologists to improve prognosis [6].

Most RUCs occur in the renal pelvis, while a few cases are reported in the infundibulum and calyces [7]. Since UC shows exceptional potential to spread along the urinary tract, a tiny minority of RUCs present multiple intrarenal lesions with hydronephrosis [8]. Although some RUCs are centered in the renal parenchyma, they are closely related to the collecting system due to derivation from urothelial cells. Some RUCs with multiple lesions and hydronephrosis are similar to RCCs with cystic-dominant lesions [9], and RUC lesions presenting as solid renal masses may be confused with RCC with collecting system invasion (CSI) at the corresponding location [9–11]. European Association of Urology Guidelines recommends computed tomography (CT) examination to diagnose UUTUC [6]. However, there is still a challenge in diagnosing preoperative CT examinations considering similar tumor location and morphology between RUC mimicking RCC and ccRCC with CSI [12, 13].

Some studies have been conducted to distinguish RUC mimicking RCC from RCC. Raza et al. defined central RCC extensively as lesions involving endophytic and some exophytic renal masses and included more subtypes of RCC except for ccRCC in differential diagnosis [14]. Bata et al. only compared the CT values between RUC and ccRCC without considering the CT-derived morphological characteristics [13]. Chen et al. constructed an easy-to-reach differential model for distinguishing exophytic/endophytic RUC mimicking RCC from ccRCC, respectively [15, 16]. Previous studies explored the diagnostic potential of CT features in identifying and assessing the prognosis of ccRCCs with CSI [14, 17]. However, previous studies were limited in applying clinical characteristics such as symptoms and history to differentiate between RUC and ccRCC. Neither Raza nor Bata et al. determined the correlation between clinical data and CT in identifying RUC and ccRCC [12, 13]. Zhu and Ding et al. only described the clinical and CT features of RUC without comparing RUC and RCC [18, 19]. Clinical characteristics have yielded reference value for diagnosis, such as smoking history, presence of nephrolith, hematuria, and flank pain [20–25]. Combining clinical characteristics and CT findings might increase differential abilities between RCC and UUTUC.

To our knowledge, no previous studies have combined clinical and CT features in the differential diagnosis of RUC, mimicking RCC and ccRCC with CSI. In this study, we aimed to explore the diagnostic potential of the CT findings and clinical characteristics in distinguishing RUC from ccRCC.

## Materials and methods

### Patients

**Fig. 1:**
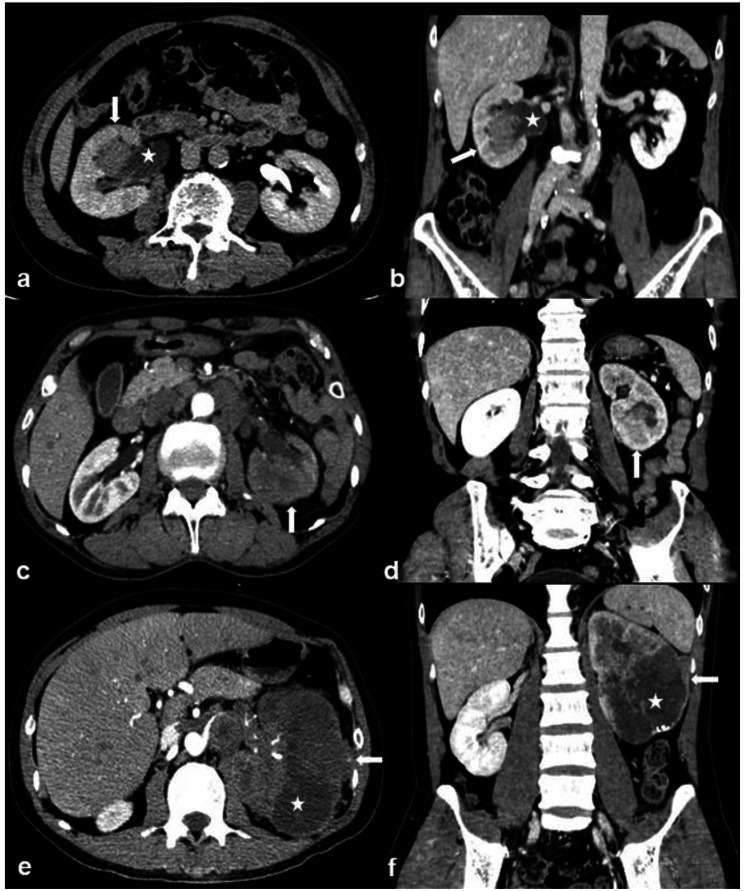
Definition of RUC mimicking RCC. (**A**) and (**B**): A 63-year-old man with RUC mimicking RCC. An endophytic nodule (white arrow) in the right renal pelvis with mild and homogeneous enhancement and an unclear boundary and micro-lobular. (**C**) and (**D**): A 69-year-old man with RUC mimicking RCC. An exophytic mass (white arrow) in the left renal parenchyma with mild and homogeneous enhancement and a vague margin. (**E**) and (**F**): A 46-year-old man with RUC mimicking RCC. A cyst-solid lesion occupied the middle and lower part of the left kidney. There were multi-neoplasms (white arrow) with uneven density, different sizes, unclear boundaries, and hydronephrosis (white star)

**Fig. 2:**
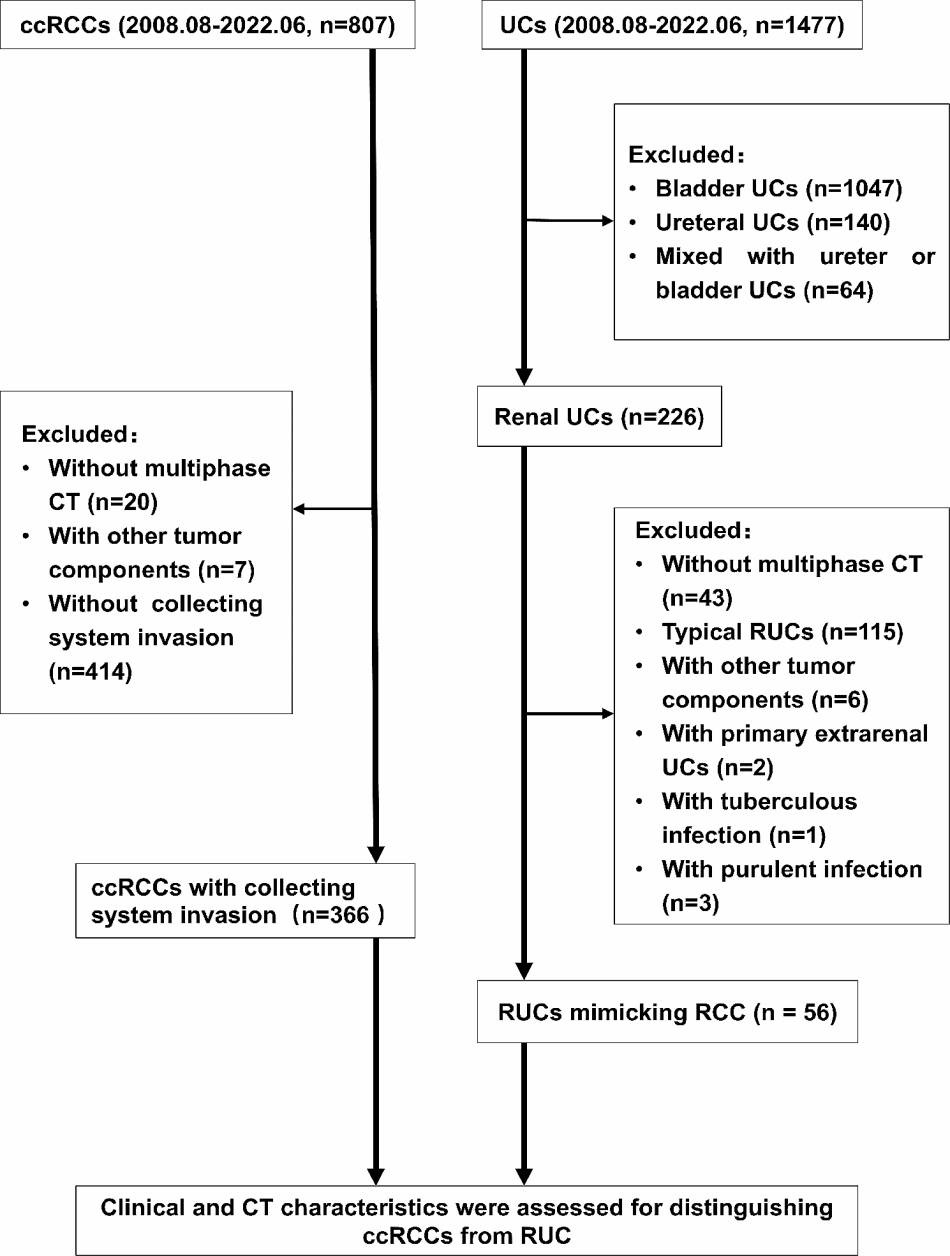
Flow chart for patient selection. (ccRCC, clear cell renal cell carcinoma; CT, computed tomography; RUC, renal urothelial carcinoma; UC, urothelial carcinoma)

The data from patients who underwent surgery and/or biopsy in our hospital from August 2008 to June 2022, including 226 RUCs and 807 ccRCCs, were collected and analyzed retrospectively. All patients underwent multiphase CT scanning within one week before biopsy and/or surgery. Patients who met the definitions of RUC mimicking RCC were included in this investigation. In the present study, RUC mimicking RCC was defined as Fig. 1: ① A nodular neoplasm (may have micro-lobular, but has no deep lobulation, generally oval) in the renal pelvis without thickening of the renal sinus wall, partially filled with the tumor in the renal sinus; ② A mass in the renal parenchyma involving and/or distorting the collecting system; ③ Cyst-solid lesion partially occupied kidney. Patients who met the definitions of ccRCC with CSI were involved (A tumor causing a filling defect in EP; in contact with the collecting system on CT image; and/or separated from the collecting system) [17]. Patients were excluded if: (1) RUCs accompanied by lesions of the ureter and/or bladder; (2) Patients mixed with other neoplastic components; (3) Patients with tuberculous/purulent infections in the urinary system. A total of 56 RUCs and 366 ccRCCs were included ultimately. The flowchart is shown in Fig. 2.

### Clinical and pathological data

Clinical data were collected using the hospital information system (HIS) (DHC Software Co, China), including sex, age, smoking history, kidney stone history, flank pain, and hematuria. Patients with a smoking history for more than 20 years and no less than 20 cigarettes per day were identified as having a smoking history [23]. Patients with kidney stones for more than five years were considered to have a positive nephrolith history [25], and cases with small asymptomatic calculi were not considered positive. Cases with positive urine routine occult blood and without visible blood were considered to have microscopic hematuria.

Pathological information was obtained from the picture archiving and communication system (PACS) (Shenzhen Annet Information System Co. Ltd, China). Two pathologists with over ten years of experience reviewed histology slides from all eligible patients to determine pathologic diagnosis, combining microscopic and immunohistochemical examination results. Another senior pathologist blinded to the information made the ultimate judgment for cases without consensus.

### Computed tomography scanning

Patients underwent multiphase CT scanning, including unenhanced phase (UP) scanning and contrast enhancement scanning of the corticomedullary phase (CP), nephrographic phase (NP), and excretory phase (EP) using 16-slice, 64-slice, 128-slice, and 256-slice spiral CT scanners (Light Speed VCT and Revolution, GE Healthcare, US; SOMATOM sensation 16, SOMATOM Definition Flash, and SOMATOM Force, Siemens Healthcare, Germany). The scanning area was from the top of the diaphragm to the iliac wing level using scanning parameters: tube voltage, 120 kV; tube current, 250–300 mA; and slice thickness, 2.5 or 5 mm. The contrast agent (Iopamiro, 300 mgI/mL; Shanghai Bracco Sine Pharmaceutical Corp. Ltd., China) was injected after plain scanning using a high-pressure syringe around the median cubital vein, with an injection flow rate of 3 mL/s. Contrast-enhanced CT phases were performed at 25–30 s for CP, 75–85 s for NP, and 210–280 s for EP.

### Imaging analysis

All CT images were acquired from the PACS of our hospital. Regarding reference from the report template for indeterminate renal masses [26], the CT features were analyzed, including size, location, infiltrative growth pattern, nephrolith, hydronephrosis, necrosis, and enhancement pattern.

The maximum tumor diameter was measured from the axial direction. The criteria of tumor location developed by Gervais et al. defined endophytic (exophytic) renal tumors as tumors centrally located in the renal pelvis (renal parenchyma or perinephric fat) [2]. Tumor growth patterns were categorized as bean-shaped (infiltrative tumor growth using renal parenchyma as scaffold) and ball-shaped (expansive growth dominant) [27]. The enhancement patterns were composed of hypo-enhancement defined as 0–40 Hounsfield units (HU) of absolute enhancement in the CP and hyperenhancement defined as ≥70 HU of absolute enhancement in the CP [28]. The heterogeneous enhancement was defined as a no-enhanced or low-attenuation area through visual assessment in the soft-tissue window (300/40) [29, 30]. The average CT value in each phase was evaluated from three circular regions of interest with the same size depicted in the most homogeneous area, avoiding blood vessels, calcification, stones, cystic necrosis, and adjacent renal parenchyma [13]. Attenuation values were described in HU. Two radiologists (with more than ten years of experience in abdominal CT diagnosis) assessed CT features, blinded to clinical and pathological information. Another senior radiologist determined the ultimate CT findings if there were differences from the initial screening.

### Statistical analyses

Statistical analyses were performed using IBM SPSS software for Windows version 22.0 (IBM Corp., US). The agreements between the two readers were evaluated using the kappa and interclass correlation coefficients (Supplementary Tables 1 and 2). The categorical variables were presented as the count of each subgroup and corresponding proportion, n (%). The data distribution for normality was evaluated using the Kolmogorov-Smirnov test. The continuous variables were described as median (range) for non-normalized variables and mean ± standard deviation for normalized variables. The data comparison was performed by Student’s t-test or Mann-Whitney U test for continuous variables, Chi-square test, or Fischer’s exact test for categorical variables. The clinic and CT features of ccRCC and RUC were summarized in Supplementary Tables 3 and 4.

The discriminative significance of each variable was evaluated using the receiver operating characteristic (ROC) curve analysis. The significant variables for classifying RUC and ccRCC were selected according to the following steps. First, variables with an area under the curve (AUC) less than 0.7 in the ROC curve analysis were excluded, which is considered low accuracy, according to Swets [31]. Next, variables with statistical significance were analyzed using univariate logistic regression for associations with RUC. Then, variables with weak associations and odd ratios (ORs) close to 1 in the univariate logistic regression analysis were excluded to avoid overfitting the predictive model [32–34]. Finally, the statistically significant variables in multivariate logistic regression were selected as independent predictors to construct the integrated predictive model (Supplementary Eq. 1) [35]. The Hosmer-Lemeshow test was used to calibrate the statistical significance of the model. The ROC curves were drawn based on independent predictors and the predictive model to assess the performance of CT and clinical characteristics in distinguishing RUC from ccRCC. A *p*-value less than 0.05 was considered statistically significant.

## Results

All kappa and interclass correlation coefficients were identified as > 0.8, indicating excellent agreement between the two readers. All continuous variables were non-normally distributed except UP and CP (Supplementary Tables 1, 2).

The clinical characteristics of eligible patients, including 56 patients with RUC (46 males and ten females) and 366 patients with ccRCC (262 males and 104 females), are summarized in Supplementary Table 3. The median age was 65.50 (range: 56.25–69.75) in the RUC group and 53.50 (range: 42.25–62.50) in the ccRCC group (*p* < 0.001). The proportion of flank pain was different between RUC (38 patients, 67.9%) and ccRCC (148 patients, 40.5%) (*p* < 0.001). There was a statistically significant difference in the history of kidney stones (*p* < 0.001) between RUC (18 patients, 32.2%) and ccRCC (25 patients, 6.8%). The distribution of hematuria classification was also different between RUC (29 patients with gross hematuria and 18 patients with microscopic hematuria) and ccRCC (75 [20.5%] patients with gross hematuria and 119 [32.5%] patients with microscopic hematuria) (*p* < 0.001).

The results of CT features are summarized in Supplementary Table 4. No differences were observed in the side, size, and renal sinus invasion between RUC and ccRCC. The location, infiltrative growth pattern, and tumor shape were statistically different (*p* < 0.001) between RUC and ccRCC. Preserving reniform contour, perinephric stranding, and hydronephrosis were more common in the RUC group than in the ccRCC group (*p* < 0.001). Pseudo-capsule sign (*p* < 0.001), heterogeneous enhancement (*p* < 0.001), and calcification (*p* = 0.044) were less common in the RUC group than in the ccRCC group (*p* < 0.001). There were higher proportions of renal calculus (*p* < 0.001), renal vein invasion (*p* < 0.001), lymphatic node metastasis (*p* < 0.001), and distant metastasis (*p* = 0.011) in RUC compared with those in ccRCC. The median/mean CT values of RUC in the multiphase scan were significantly lower than those of ccRCC (*p* < 0.001).

**Fig. 3:**
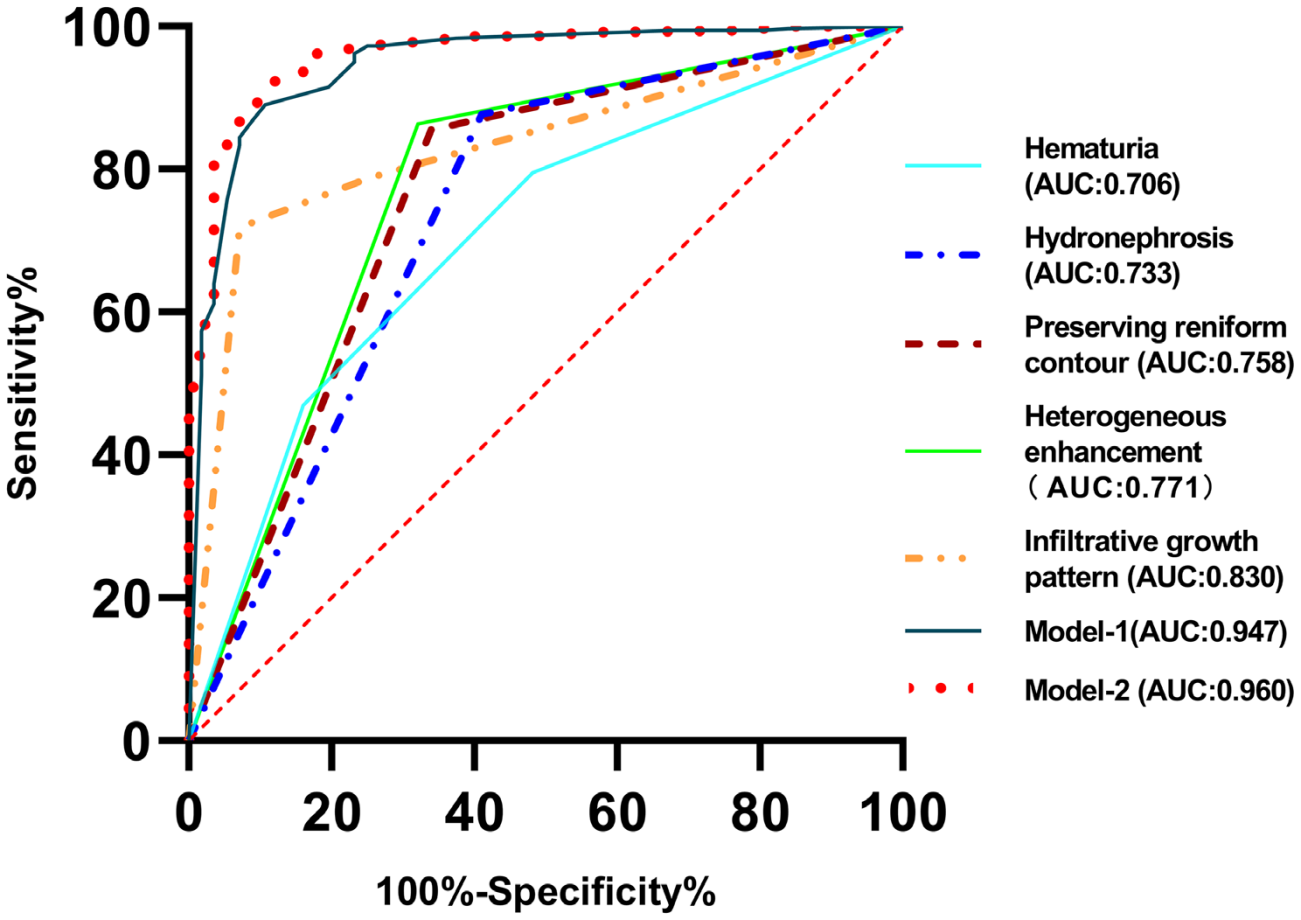
Diagnosis performance of the CT and clinical characteristics for differentiation between RUC and ccRCC. AUC, area under the curve; ccRCC, clear cell renal cell carcinoma; RUC, renal urothelial carcinoma. Model-1 = 1/[1 + e ^– (2.765 Heterogeneous enhancement − 2.504 Bean shape − 0.004 Cyst−solid appearance − 2.423 Hydronephrosis – 1.589 Preserving reniform contour + 0.451)^]. Model-2 = 1/[1 + e ^– (3.094 Heterogeneous enhancement − 2.460 Bean shape − 0.029 Cyst−solid appearance − 2.528 Hydronephrosis – 1.229 Preserving reniform contour − 2.241 Gross hematuria – 1.375 Microscopic hematuria + 1.220)^]

Variable assignments before logistic regression analysis are summarized in Supplementary Table 5. The logistic regression results are shown in Table 1. Age (OR = 1.086, *p* < 0.001), CP (OR = 0.914, *p* < 0.001), NP (OR = 0.938, *p* < 0.001), and EP (OR = 0.955, *p* < 0.001) were excluded due to their weak associations. The flank pain (AUC = 0.637, *p* < 0.001), perinephric stranding (AUC = 0.635, *p* < 0.001), history of kidney stones (AUC = 0.627, *p* = 0.002), lymphatic node metastasis (AUC = 0.627, *p* = 0.002), tumor shape (AUC = 0.626, *p* = 0.002), renal calculus (AUC = 0.616, *p* = 0.005), renal vein invasion (AUC = 0.587, *p* = 0.036), and distant metastasis (AUC = 0.558, *p* = 0.162) were then excluded because of low accuracy (Table 2). The infiltrative growth pattern, pseudo-capsule sign, hydronephrosis, heterogeneous enhancement, preserving reniform contour, location, and hematuria were ultimately included in the multivariate logistic regression analysis (Table 1). The infiltrative growth pattern, hydronephrosis, heterogeneous enhancement, preserving reniform contour, and hematuria were identified as independent predictors (*p* < 0.05) to construct the predictive models (model-1 and model-2) in distinguishing RUC from ccRCC (Table 1). According to Supplementary Tables 6, 7, the predictive probabilities were calculated using the formula: *p*1 = 1/[1 + e ^– (2.765 Heterogeneous enhancement − 2.504 Bean shape − 0.004 Cyst−solid appearance − 2.423 Hydronephrosis – 1.589 Preserving reniform contour + 0.451)^]. *p*2 = 1/[1 + e ^– (3.094 Heterogeneous enhancement − 2.460 Bean shape − 0.029 Cyst−solid appearance − 2.528 Hydronephrosis – 1.229 Preserving reniform contour − 2.241 Gross hematuria – 1.375 Microscopic hematuria + 1.220)^]. The Hosmer-Lemeshow test revealed the consistency between the calculated probabilities and actual observations (*p*1 = 0.799, *p*2 *=* 0.634). The AUCs of model-1 and model-2 were 0.947 and 0.960 with a sensitivity of 89.07% and 92.08% and specificity of 89.29% and 89.29%, respectively (Table 2) (Fig. 3). The performances of model-1(construction by using CT characteristics only) and model-2 (construction using Clinical and CT characteristics) were both better than that of the predictors. Furthermore, the performance of model 2 was the best.

**Table 1:** Logistic regression analysis for Clinical-CT characteristics of RUC and ccRCC

Variable	Univariate logistic regression			Multivariate logistic regression		
	*p*	OR	95%CI	*p*	OR	95%CI
IGP						
Bean shape	< 0.001	0.028	0.010–0.081	0.001	0.114	0.031–0.420
CsA	0.479	0.743	0.326–1.693	0.825	0.861	0.228–3.258
Ball shape		Ref. (1.000)			Ref. (1.000)	
PcS	< 0.001	14.034	4.973–39.604	0.293	2.074	0.532–8.085
No		Ref. (1.000)			Ref. (1.000)	
Hydronephrosis	< 0.001	0.098	0.053–0.181	< 0.001	0.1	0.032–0.319
No		Ref. (1.000)			Ref. (1.000)	
HE	< 0.001	13.342	7.069–25.182	< 0.001	20.129	6.522–62.127
No		Ref. (1.000)			Ref. (1.000)	
PRC	< 0.001	0.087	0.047–0.162	0.047	0.345	0.120–0.987
No		Ref. (1.000)			Ref. (1.000)	
Hematuria						
GH	< 0.001	0.135	0.061-0.300	0.001	0.113	0.031–0.409
MH	0.005	0.391	0.203–0.753	0.009	0.254	0.089–0.726
No		Ref. (1.000)			Ref. (1.000)	
Location						
Endophytic	< 0.001	0.129	0.071–0.236	0.278	0.565	0.202–1.585
Exophytic		Ref. (1.000)			Ref. (1.000)	
LNM	< 0.001	0.119	0.057–0.249			
No		Ref. (1.000)				
Flank Pain	< 0.001	0.322	0.177–0.585			
No		Ref. (1.000)				
HKS	< 0.001	0.155	0.077–0.309			
No		Ref. (1.000)				
Renal calculus	< 0.001	0.209	0.108–0.407			
No		Ref. (1.000)				
RVI	0.001	0.315	0.162–0.613			
No		Ref. (1.000)				
PS	< 0.001	0.322	0.181–0.572			
No		Ref. (1.000)				
Tumor shape						
Irregular	0.001	0.301	0.151–0.601			
Regular		Ref. (1.000)				
DM	0.013	0.400	0.194–0.826			
No		Ref. (1.000)				
Age	< 0.001	1.086	1.055–1.118			
UP	0.413	0.982	0.940–1.026			
CP	< 0.001	0.914	0.893–0.936			
NP	< 0.001	0.938	0.919–0.958			
EP	< 0.001	0.955	0.935–0.974			

ccRCC, clear cell renal cell carcinoma; CI, confidence interval; CP, corticomedullary phase; CsA, cyst-solid appearance; DM, distant metastasis; GH, gross hematuria; HE, heterogeneous enhancement; HKS, history of kidney stones; IGP, infiltrative growth pattern; LNM, lymphatic node metastasis; MH, microscopic hematuria; PcS, pseudo-capsule sign; PRC, preserving reniform contour; PS, perinephric stranding; RUC, renal urothelial carcinoma.; RVI, renal vein invasion

As triple categorical variables (hematuria and infiltrative growth pattern), their OR values were obtained by comparing gross hematuria (bean shape) and microscopic hematuria (cyst-solid appearance) with No (ball shape), respectively. The reference category was No (ball shape). In the table, we expressed the two remaining categories as gross hematuria (bean shape) and microscopic hematuria (cyst-solid appearance)

**Table 2:** ROC curves of the clinical-CT characteristics for diagnosis RUC or ccRCC

Variable	AUC	*p*	95%CI	Sensitivity%	Specificity%
Infiltrative growth pattern	0.830	< 0.001	0.780–0.880	71.86	92.86
Heterogeneous enhancement	0.771	< 0.001	0.696–0.846	86.34	67.86
Preserving reniform contour	0.758	< 0.001	0.682–0.834	85.52	66.07
Hydronephrosis	0.733	< 0.001	0.653–0.814	87.70	58.93
Pseudo-capsule	0.724	< 0.001	0.665–0.783	51.91	92.86
Location	0.720	< 0.001	0.641–0.780	83.33	60.71
Hematuria	0.706	< 0.001	0.634–0.778	79.51	51.79
Flank pain	0.637	0.001	0.560–0.714	59.56	67.86
PerinephricStranding	0.635	0.001	0.555–0.716	69.95	57.14
History of kidney stones	0.627	0.002	0.539–0.714	93.17	32.14
Lymphatic metastasis	0.627	0.002	0.539–0.715	95.08	30.36
Tumor shape	0.626	0.002	0.553–0.699	44.81	80.36
Renal calculus	0.616	0.005	0.529–0.703	90.98	32.14
Renal vein invasion	0.587	0.036	0.501–0.673	88.80	28.57
Distant metastasis	0.558	0.162	0.473–0.643	90.16	21.43
Model-1	0.947	< 0.001	0.914–0.980	89.07	89.29
Model-2	0.960	< 0.001	0.935–0.985	92.08	89.29

CI, confidence interval; CP, corticomedullary phase; ccRCC, clear cell renal cell carcinoma; RUC, renal urothelial carcinoma

Model-1 = 1/[1 + e ^– (2.765 Heterogeneous enhancement − 2.504 Bean shape − 0.004 Cyst−solid appearance − 2.423 Hydronephrosis – 1.589 Preserving reniform contour + 0.451)^]. Model-2 = 1 / [1 + e ^– (3.094 Heterogeneous enhancement − 2.460 Bean shape − 0.029 Cyst−solid appearance − 2.528 Hydronephrosis – 1.229 Preserving reniform contour − 2.241 Gross hematuria – 1.375 Microscopic hematuria + 1.220)^

## Discussion

To our knowledge, no studies have defined and classified RUC as mimicking RCC. Aside from that, this study provided a new approach for diagnostic reference. The model-1’s performance itself was better than the predictors’. After adding the clinical characteristic to construct the predictive model, the performance of model 2 was further improved. The present predictive model could imply the presence of RUCs before surgery. The predictors from clinical data and CT findings assisted in distinguishing RUC from ccRCC.

**Fig. 4:**
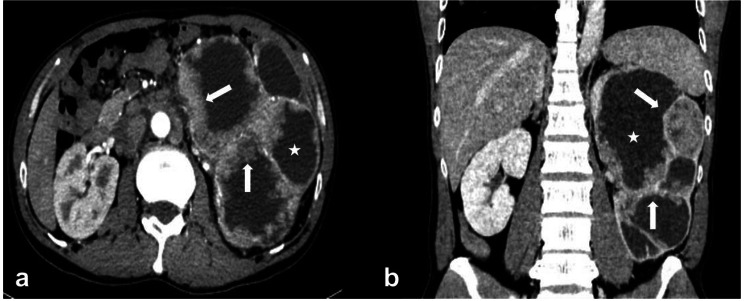
A 45-year-old man with RUC mimicking RCC. (**A**) and (**B**): The left kidney was present with a cyst-solid lesion due to hydronephrosis, leading renal pelvises and calyces to dilate, with uneven and irregular thickening of the renal sinus walls and multiple-sized tumors (white arrows)

**Fig. 5:**
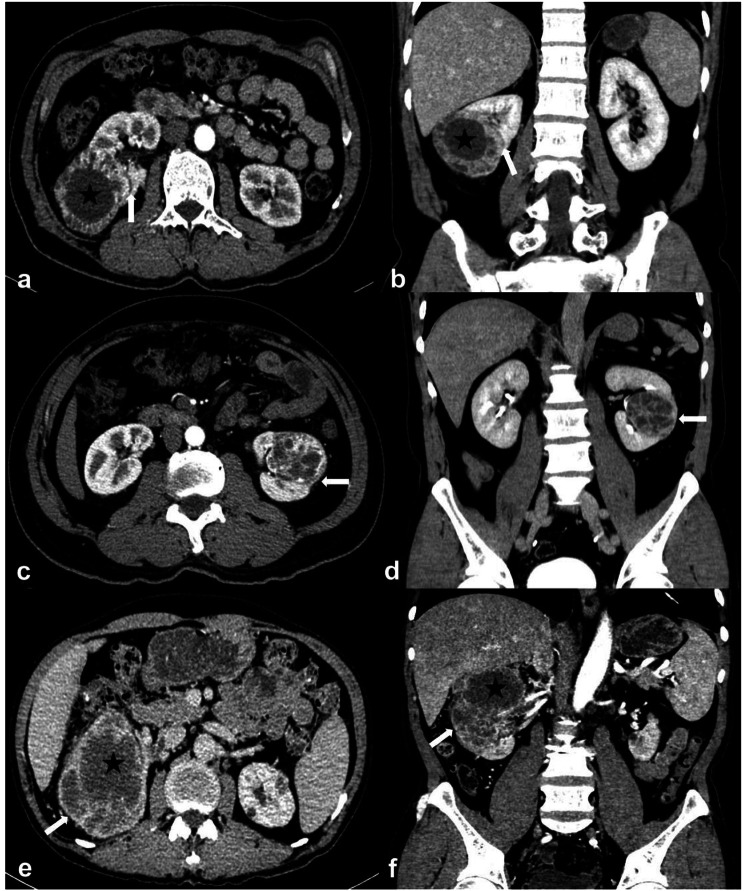
Cystic-solid ccRCC with collecting system invasion. (**A**) and (**B**): A 59-year-old man with a ccRCC invading the collecting system. A heterogeneous enhanced high attenuation renal mass in the left renal pelvis, with cystic components dominant (black star) and pseudo-capsule sign (thin white arrow). (**C**) and (**D**): A 62-year-old man suffering from ccRCC with collecting system invasion. An exophytic cystic-dominant heterogeneous hyper-enhanced renal mass in the right kidney, with reniform deformation. The tumor was expansive growth with a clear boundary and pseudo-capsule sign (thin white arrow). (**E**) and (**F**): A 53-year-old man suffering from ccRCC with collecting system invasion. An endophytic mass in the right kidney (white arrow), with cystic component (black star), heterogeneous hyper-enhanced solid component, and poorly defined interface

This study illustrated that RUC was more likely to present infiltrative growth using renal parenchyma as the frame (bean shape) (Figs. 1c and d and 4). In contrast, the dominantly expanded tumor (ball shape) was more common in ccRCC (Fig. 5a-d). These results were consistent with previous reports [12, 18, 19]. The cyst-solid lesions were found uncommon in both RUC and ccRCC. Since multi-RUC with hydronephrosis displayed a cyst-solid lesion of the kidney, it was essential to distinguish it from the cystic-dominant ccRCC. The cyst-solid lesions of multi-RUCs were caused by multiple tumors, renal sinus walls, and hydronephrosis (Fig. 1e, f, and Fig. 4), which were also found by Prando et al. [8]. The cyst-solid ccRCC was composed of necrosis, cystic degeneration, and solid component, usually without hydronephrosis (Fig. 5). Most cyst-solid ccRCCs were exophytic and easy to be distinguished from RUCs (Fig. 5a, b). Endophytic cyst-solid ccRCCs having clear boundaries with heterogeneous solid components and noticeable enhancement were also easily distinguished from RUC (Fig. 5c, d). However, it was challenging to distinguish multi-RUCs with hydronephrosis from other endophytic cyst-solid ccRCCs (Figs. 1e and f and 5 e, f).

**Fig. 6:**
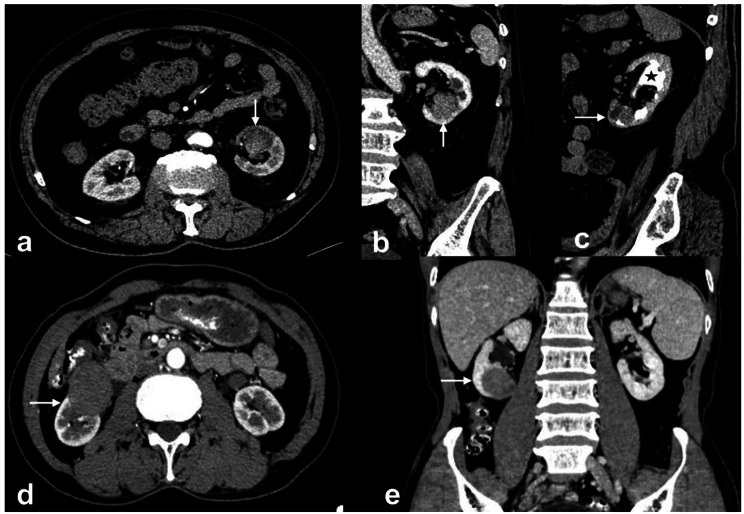
RUC with pseudo-capsule sign. (**A**) and (**B**): A 66-year-old man with RUC mimicking RCC. An endophytic mass in the left renal pelvis with mild and homogeneous enhancement, pseudo-capsule (thin white arrow), and hydronephrosis (black star). (**C**) and (**D**): A 66-year-old man with RUC mimicking RCC. A mild and homogeneously enhanced exophytic mass in the right kidney, with clear boundary, pseudo-capsule (thin white arrow), and hydronephrosis (white star)

In addition, due to infiltrative growth of RUC in the renal parenchyma, the renal contour, even for exophytic RUC, was maintained (Fig. 1c, d), whereas the ccRCC commonly deformed renal contour due to expansive growth (Fig. 5a, b). RUC was more prone to hydronephrosis due to the close relationship with the collecting system (Figs. 1 and 4, and 6), which was consistent with findings from Prando et al. [8] and Chen et al. [15, 16]. In this study, the attenuation of RUC (Figs. 1, 4 and 6) was lower than that of ccRCC (Fig. 5), with less heterogeneous enhancement as reported [12, 13]. This might attribute to the poor blood supply and limited possibilities of necrosis in RUC. Some scholars believed that necrosis and heterogeneous enhancement increased with larger ccRCC [30, 36, 37]. Additionally, the present study found that patients with RUC were more susceptible to hematuria due to its high possibility of urothelium invasion.

Although some features were excluded in constructing the predictive model, such as age, CP, NP, and EP, to avoid overfitting, location and pseudo-capsular sign were excluded for predictor screening, they still represented positive function in differentiating RUC from ccRCC. In this study, patients with ccRCC were younger than those with RUC, similar to results from previous reports [12, 13, 15, 16]. Different from the report of Bata et al. (Only in CP and NP, the attenuation of ccRCC was significantly higher than RUCs’) [13], the attenuation of ccRCC (Fig. 5) was significantly higher than that of RUC (Figs. 1 and 4, and 6) for each dynamic contrast-enhanced phase in our study. RUC was more likely to occur in the renal pelvis than in the calyces (Fig. 1a, b), and ccRCC were exophytic renal tumors for most cases (Fig. 5a, b), which were consistent with previous reports [12]. It is worth mentioning that pseudo-capsule signs were noted in 4 RUCs in this study (Fig. 6). Chen et al. reported a pseudo-sign in an RUC case with a kidney rupture [15]. Moreover, Chen et al. have also reported a pseudo-capsule sign in endophytic RUC [16]. The pseudo-capsule sign for the endophytic RUC case differed from that of RCC (Fig. 5a-d). However, this sign for the exophytic low-grade RUC might be similar to that of RCC (Fig. 6d, e). The former RUC cases were related to the compression of renal pelvis fat. The latter RUC cases might be ischemic necrosis led by the deposition of fibrous tissue after compression (Fig. 6a, b, c) [38].

Some characteristics of RUCs in our study were different from previous literature. The presence of nephrolith, hydronephrosis, flank pain, hematuria, and cyst-solid lesions was not found in previous research articles [12, 18, 19], except Chen et al. [15, 16]. These consistencies might be attributed to different populations, lifestyles, and large lesions analyzed in this study. Besides, the incidence of CSI in our ccRCC seemed higher than those of reports. One reason may be that the previous reports are only about the incidence of CSI in RCC [14, 17]. RCC includes many histological types except ccRCC. Furthermore, the population in our study may be different from others.

This study had certain limitations. First, the sample size of RUC mimicking RCC remained small compared to that of ccRCC, and an external validation cohort was lacked. Second, it only demonstrated the clinical and CT characteristics of RUC and ccRCC without including other RCC subtypes in this study. Third, this study only focused on the diagnostic task of ccRCC and RUC without obtaining direct data for prognostic analysis. Fourth, limitations of human interpretation. In the next step of this study, we would prepare an external validation cohort with many cases. Furthermore, it is crucial to construct predictive models with radiomics or artificial intelligence.

## Conclusion

The predictive model-2 incorporating CT and clinical characteristics showed extraordinary differential abilities to distinguish RUC mimicking RCC from ccRCC with CSI. A renal tumor with those features of infiltrative growth dominance, hydronephrosis, homogeneous enhancement, preserving reniform contour, and hematuria inclined to RUC, and if old patients with endophytic renal mass and without a pseudo-capsule sign that might be considered as RUC.

### Electronic supplementary material

Below is the link to the electronic supplementary material.

Supplementary Material 1

## Data Availability

The datasets used and/or analyzed during the current study are available from the corresponding author upon reasonable request.

## Abbreviations

AUC: area under the curve
ccRCC: clear cell renal cell carcinoma
CI: confidence interval
CP: corticomedullary phase
CT: computed tomography
CSI: collecting system invasion
EP: excretory phase
HIS: hospital information system
HU: Hounsfield units
NP: nephrographic phase
OR: odds ratio
PACS: picture archiving and communication system
RCC: renal cell carcinoma
ROC: receiver operating characteristic
RUC: renal urothelial carcinoma
UC: urothelial carcinoma
UP: unenhanced phase
UUTUC: Upper urinary tract urothelial carcinoma
