# Supplementary material for: CT findings and clinical characteristics in distinguishing renal urothelial carcinoma mimicking renal cell carcinoma from clear cell renal cell carcinoma
Source: BMC Urol. 2024 Jan 3;24:4. doi: 10.1186/s12894-023-01393-5 (PMC10765735; doi:10.1186/s12894-023-01393-5)
Supplement: Supplementary file 1 — Supplementary Material 1 [file 12894_2023_1393_MOESM1_ESM.docx]

**Supplementary Table 1**. The results for interclass correlation coefficient of the two readers’ agreement

| **Characteristics** | **ICC** | **95%CI** | ***p*** |
| --- | --- | --- | --- |
| Size | 0.885 | 0.862-0.904 | <0.001 |
| UP | 0.827 | 0.794-0.855 | <0.001 |
| CP | 0.832 | 0.800-0.859 | <0.001 |
| NP | 0.905 | 0.886-0.921 | <0.001 |
| EP | 0.866 | 0.840-0.888 | <0.001 |

**Supplementary Table 2**. The results for kappa test of the two readers’ agreement

| **Characteristics** | **Kappa** | **95%CI** | ***p*** |
| --- | --- | --- | --- |
| Location | 0.883 | 0.834-0.932 | <0.001 |
| Renal Sinus invasion | 0.833 | 0.780-0.886 | <0.001 |
| Infiltrative growth pattern | 0.866 | 0.821-0.911 | <0.001 |
| Tumor shape | 0.810 | 0.755-0.865 | <0.001 |
| Pseudo-capsule sign | 0.845 | 0.794-0.896 | <0.001 |
| Preserving reniform contour | 0.872 | 0.813-0.931 | <0.001 |
| Perinephric stranding | 0.861 | 0.810-0.912 | <0.001 |
| Hydronephrosis | 0.960 | 0.925-0.995 | <0.001 |
| Heterogeneous enhancement | 0.819 | 0.754-0.884 | <0.001 |
| Calcification | 0.969 | 0.940-0.998 | <0.001 |
| Calculus | 0.947 | 0.900-0.994 | <0.001 |
| Renal vein invasion | 0.871 | 0.804-0.938 | <0.001 |
| Lymphatic metastasis | 0.850 | 0.764-0.936 | <0.001 |
| Distant metastasis | 0.944 | 0.895-0.993 | <0.001 |

**Supplementary Table 3.** Clinical data for RUC and ccRCC

| **Variable** | **RUC（n=56）** | **ccRCC=（n=366）** | ***p*** |
| --- | --- | --- | --- |
| Smoking history | 18 (32.14) | 91 (24.86) | 0.246 |
| Flank pain | 38 (67.86) | 148 (40.44) | <0.001 |
| History of kidney stones | 18 (32.14) | 25 (6.83) | <0.001 |
| Hematuria |  |  | <0.001 |
| Gross hematuria | 29 (51.79) | 75 (20.49) |  |
| Microscopic hematuria | 18 (32.14) | 119 (32.51) |  |
| Sex |  |  | 0.097 |
| Male | 46 (82.14) | 262 (71.58) |  |
| Female | 10 (17.86) | 104 (28.42) |  |
| Age(year) | 65.50 (56.25-69.75) | 53.50 (42.25-62.50) | <0.001 |

ccRCC, clear cell renal cell carcinoma; RUC, renal urothelial carcinoma.

**Supplementary Table 4.** CT characteristics for RUC and ccRCC

| **Variable** | **RUC（n=56）** | **ccRCC（n=366）** | ***p*** |
| --- | --- | --- | --- |
| Side |  |  | 0.850 |
| Left | 30 (53.57) | 201 (54.92) |  |
| Right | 26 (46.43) | 165 (45.08) |  |
| Size(cm) | 51.01 (40.58-66.56) | 42.40 (32.15-56.77) | 0.308 |
| Location |  |  | <0.001 |
| Endophytic | 34 (60.71) | 61 (16.67) |  |
| Exophytic | 22 (39.29) | 305 (83.33) |  |
| Renal sinus invasion | 33 (58.93) | 166 (45.36) | 0.058 |
| Infiltrative growth pattern |  |  | <0.001 |
| Bean shape | 42 (75.00) | 78 (21.31) |  |
| Cyst-solid appearance | 10 (17.86) | 25 (6.83) |  |
| Ball shape | 4 (7.14) | 263 (71.86) |  |
| Tumor shape |  |  | <0.001 |
| Irregular | 45 (80.36) | 202 (55.19) |  |
| Regular | 11 (19.64) | 164 (44.81) |  |
| Pseudo-capsule sign | 4 (7.14) | 190 (51.91) | <0.001 |
| Preserving reniform contour | 37 (66.07) | 53 (14.48) | <0.001 |
| Perinephric stranding | 32 (57.14) | 110 (30.05) | <0.001 |
| Hydronephrosis | 33 (58.93) | 45 (12.30) | <0.001 |
| Heterogeneous enhancement | 18 (32.14) | 316 (86.34) | <0.001 |
| Calcification | 5 (8.93) | 74 (20.22) | 0.044 |
| Renal Calculus | 18 (32.14) | 33 (9.02) | <0.001 |
| Renal vein invasion | 16 (28.57) | 41 (11.20) | <0.001 |
| Lymphatic node metastasis | 17 (30.36) | 18 (4.92) | <0.001 |
| Distant metastasis | 12 (21.43) | 36 (9.84) | 0.011 |
| UP(HU) | 36.57±4.03 | 37.34±6.91 | 0.001 |
| CP(HU) | 63.18±13.03 | 108.81±35.91 | <0.001 |
| NP(HU) | 77.74（66.39-85.30） | 90.41（78.20-106.25） | <0.001 |
| EP(HU) | 66.88（57.06-73.58） | 72.98（65.68-97.79） | <0.001 |

ccRCC, clear cell renal cell carcinoma; CP, corticomedullary phase; EP, excretory phase; HU, Hounsfield unit; NP, nephrogenic phase; RUC, renal urothelial carcinoma; UP, unenhanced phase.

**Supplementary Table 5.** Variable assignments before logistic regression analysis

| DM |  | Yes | No |
| --- | --- | --- | --- |
| LNM |  | Yes | No |
| RVI |  | Yes | No |
| RC |  | Yes | No |
| Calcification |  | Yes | No |
| HE |  | Yes | No |
| Hydronephrosis |  | Yes | No |
| PS |  | Yes | No |
| PRC |  | Yes | No |
| PcS |  | Yes | No |
| TS |  | Yes | No |
| IGP |  | Yes | No |
| Location |  | Endophytic | Exophytic |
| Hematuria | GH | MH | NO |
| HKS |  | Yes | No |
| Assignment | 2 | 1 | 0 |

ccRCC, clear cell renal cell carcinoma; RUC, renal urothelial carcinoma; FP, flank pain; GH, gross hematuria; HE, heterogeneous enhancement; IGP, infiltrative growth pattern; MH, microscopic hematuria; PcS, pseudo-capsule sign; RC, renal calculus; TS, tumor shape; PRC, preserving reniform contour; PS, perinephric stranding; RVI, renal vein invasion; LNM, lymphatic node metastasis; DM, distant metastasis.

**Supplementary Table 6.** Construction logistic regression analysis predictive model for distinguishing RUC from ccRCC by using CT characteristic only

| Variable | ß | S.E, | p | OR | 95%CI |
| --- | --- | --- | --- | --- | --- |
| Infiltrative growth pattern |  |  |  |  |  |
| Bean shape | -2.504 | 0.607 | <0.001 | 0.082 | 0.025-0.269 |
| Cyst-solid appearance | -0.004 | 0.597 | 0.994 | 0.996 | 0.309-3.207 |
| Preserving reniform contour | -1.589 | 0.474 | 0.001 | 0.204 | 0.081-0.517 |
| Hydronephrosis | -2.423 | 0.531 | <0.001 | 0.089 | 0.031-0.251 |
| Heterogeneous enhancement | 2.765 | 0.518 | <0.001 | 15.887 | 5.760-43.819 |
| Constant | 0.451 | 0.462 | 0.329 | 1.571 |  |

ccRCC, clear cell renal cell carcinoma; CI, confidence interval; ß, regression coefficient; OR, odds ratio; SE, standard error.

**Supplementary Table 7.** Construction logistic regression analysis predictive model for distinguishing RUC from ccRCC by using clinical-CT characteristic

| **Variable** | **ß** | **S.E,** | ***p*** | **OR** | **95%CI** |
| --- | --- | --- | --- | --- | --- |
| Preserving reniform contour | -1.229 | 0.521 | 0.018 | 0.293 | 0.105-0.812 |
| Hematuria |  |  |  |  |  |
| Gross hematuria | -2.241 | 0.639 | <0.001 | 0.106 | 0.030-0.372 |
| Microscopic hematuria | -1.375 | 0.529 | 0.009 | 0.253 | 0.090-0.713 |
| Hydronephrosis | -2.528 | 0.570 | <0.001 | 0.080 | 0.026-0.244 |
| Heterogeneous enhancement | 3.094 | 0.573 | <0.001 | 22.062 | 7.181-67.781 |
| Infiltrative growth pattern |  |  |  |  |  |
| Bean shape | -2.460 | 0.641 | <0.001 | 0.085 | 0.024-0.300 |
| Cyst-solid appearance | -0.029 | 0.643 | 0.965 | 0.972 | 0.276-3.424 |
| Constant | 1.220 | 0.555 | 0.028 | 3.388 |  |

ccRCC, clear cell renal cell carcinoma; CI, confidence interval; ß, regression coefficient; OR, odds ratio; SE, standard error.

**Supplementary Equation 1.** Equation of the prediction model

Logit (*p*) = Ln [*p*/ (1-*p*)] (**1**)

*p*, the probabilistic predictive value.
